# Regulation of EGFR signalling by palmitoylation and its role in tumorigenesis

**DOI:** 10.1098/rsob.210033

**Published:** 2021-10-06

**Authors:** Yasmin A. Kadry, Jia-Ying Lee, Eric S. Witze

**Affiliations:** Department of Cancer Biology, Perelman School of Medicine, University of Pennsylvania, Philadelphia, PA 19104, USA

**Keywords:** palmitoylation, EGFR, lung, cancer, signalling, DHHC20

## Abstract

The epidermal growth factor receptor (EGFR) is an essential driver of oncogenic signalling, and EGFR inhibitors are some of the earliest examples of successful targeted therapies in multiple types of cancer. The tractability of EGFR as a therapeutic target is overshadowed by the inevitable drug resistance that develops. Overcoming resistance mechanisms requires a deeper understanding of EGFR regulation in cancer cells. In this review, we discuss our recent discovery that the palmitoyltransferase DHHC20 palmitoylates EGFR on the C-terminal domain and plays a critical role in signal regulation during oncogenesis. Inhibiting DHHC20 expression or mutating the palmitoylation site on EGFR alters the EGF-induced signalling kinetics from a transient signal to a sustained signal. The change in signalling is accompanied by a decrease in cell proliferation in multiple human cancer cell lines. Our *in vivo* studies demonstrate that ablating the gene *Zdhhc20* by CRISPR/Cas9-mediated inhibition in a mouse model of oncogenic Kras-driven lung adenocarcinoma potently inhibits tumorigenesis. The negative effect on tumorigenesis is mediated by EGFR since the expression of a palmitoylation-resistant mutant form of EGFR also inhibits Kras-driven lung adenocarcinoma. Finally, reducing EGFR palmitoylation increases the sensitivity of multiple cancer cell lines to existing inhibitors of EGFR and downstream signalling effector pathways. We will discuss the implications of these effects and strategies for targeting these new vulnerabilities.

## Graphical abstract

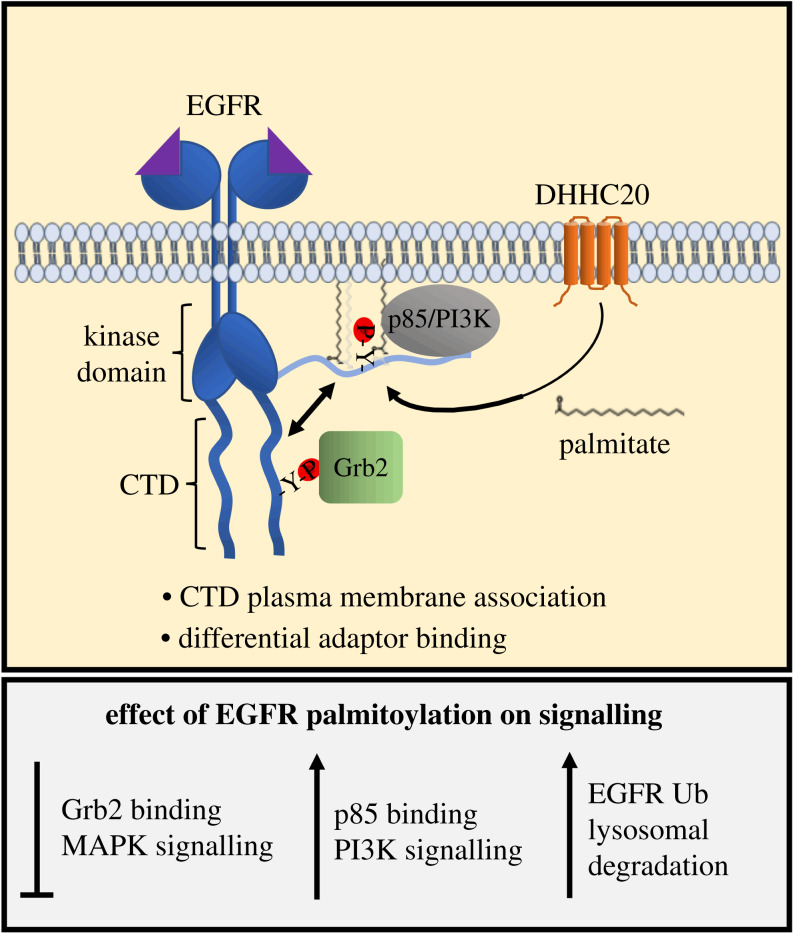

## 1. Introduction

A delicate balance between multiple convergent signalling pathways can tip the scale between homeostatic and oncogenic signalling. The cancer biology field has focused on identifying components of these pathways that can be therapeutically targeted in the clinic. Growth factor receptor signalling is a frequently dysregulated pathway in multiple cancer types. In particular, the epidermal growth factor receptor (EGFR) is one of the most frequently mutated oncogenes in lung cancer and other cancer types [1]. EGFR is a receptor tyrosine kinase that transduces signalling cascades across the plasma membrane, from the extracellular to intracellular environment [2]. The binding of extracellular ligands to the ectodomain of EGFR promotes receptor dimerization, intracellular autophosphorylation and the recruitment of effector proteins to facilitate downstream signalling [2]. Mutations and deletions in EGFR promote oncogenic signalling by enhancing the kinase activity of EGFR [3]. Although small molecules have been developed to directly target these mutations, tumours inevitably acquire resistance to these agents, most of which are tyrosine kinase inhibitors (TKIs) [4]. As a result, there has been significant interest in uncovering additional layers of EGFR signal regulation in cancer that are amenable to pharmacologic manipulation. We have recently determined EGFR is palmitoylated, and through biochemical and *in vivo* studies have demonstrated this modification inhibits receptor signalling activity and plays a critical role in cancer initiation and growth in specific cellular contexts.

## 2. Regulation of EGFR signalling by palmitoylation

### 2.1. EGFR signal regulation

EGFR is essential for normal epithelial cell function and can function as a potent oncogene when mutated [5]. EGFR is a member of the ErbB receptor tyrosine kinase family, a group of four structurally related receptor tyrosine kinases [6]. ErbB receptors are composed of an extracellular ligand-binding domain, a transmembrane domain and juxtamembrane domain that promotes plasma membrane localization, an intracellular kinase domain and a long intracellular C-terminal domain (CTD) that is largely unstructured and intrinsically disordered [2,7–9]. Under normal conditions, EGFR is found on the cell surface in a structurally compact ‘autoinhibited’ state, existing as a monomer with minimal kinase activity [10]. The binding of extracellular ligands, such as epidermal growth factor (EGF), induces conformational rearrangements in the receptor that promote homodimerization with a second EGFR monomer [10]. These rearrangements are propagated across the plasma membrane to the intracellular kinase domain, where asymmetric dimerization of the two kinase domains in a ‘head to tail’ manner promotes autophosphorylation of tyrosine residues in the CTD [11–13]. These phosphotyrosines are docking sites for cytoplasmic signalling scaffolds such as Grb2 and PI3K, which activate specific signalling cascades to promote cell growth and proliferation [14,15]. Signal termination is initiated by the recruitment of endocytic adaptors and ubiquitin ligases to the phosphorylated CTD, which promote internalization of the receptor and subsequent lysosomal degradation or recycling back to the plasma membrane [16].

While such ‘canonical’ EGFR signalling has been well studied, recently identified intricacies in EGFR regulation have shed light onto the true complexity of EGFR signalling. Upon ligand binding, EGFR monomers can also hetero-dimerize with other members of the ErbB family. Although the determinants of homodimerization versus heterodimerization are not yet well understood, it is evident that heterodimerization can tune ligand-specificity and spatio-temporal signalling [17]. These mechanisms are essential for tight regulation of EGFR signalling activity and loss of this regulation frequently leads to cell transformation and cancer.

### 2.2. EGFR mutations in cancer

EGFR has emerged as an important therapeutic target, particularly in the context of lung cancer [18]. Oncogenic mutations and alterations in EGFR allow the receptor to bypass endogenous regulatory mechanisms and signal constitutively. The EGFR kinase domain is the most frequently altered domain in the receptor, where mutations in regulatory regions of the kinase domain lead to constitutive kinase activity. The most common mutations in the kinase domain are an L858R substitution, and an in-frame exon 19 deletion (amino acids 746–750) [18]. TKIs have been developed to target these mutations, but acquired resistance is nearly inevitable, imparted by compensatory mutations in the receptor that render these agents ineffective. First-generation TKIs, such as gefitinib, which targets L858R and exon 19 deletion mutants of EGFR by reversibly competing with ATP, have been considered the first-line therapy for NSCLC patients with EGFR mutations [19]. The predominant mechanism of gefitinib resistance is a secondary T790M mutation in the kinase domain, which increases the affinity of the kinase domain for ATP over gefitinib [20]. Other resistance mechanisms include activation of downstream signalling effectors or other cell receptors. While other classes of TKIs have been developed to counteract these secondary mutations, acquired resistance remains a persistent challenge making it essential that other strategies for targeting EGFR signalling be explored.

### 2.3. EGFR is S-palmitoylated

Recently, our laboratory and others have established that EGFR is S-palmitoylated, presenting a novel mechanism of regulating EGFR signalling [21,22]. S-palmitoylation is common among many mammalian proteins and often functions to increase membrane association of cytosolic proteins. One of the most well-studied examples of this is the GTPase Ras. Specific isoforms of Ras require palmitoylation for localization to the plasma membrane, where Ras becomes activated and signals to downstream effectors in the MAPK signalling pathway regulating cell growth and proliferation [23,24]. Similarly, palmitoylation of cytosolic Src-family tyrosine kinases Yes, Lck and Fyn promote the localization of these kinases to the plasma membrane in a manner that is important for kinase function [25–27]. However, the function of palmitoylation of receptor tyrosine kinases such as EGFR, which are already localized to the plasma membrane, has not yet been established. Therefore, the finding that EGFR is palmitoylated raises several questions about the function of palmitoylation in regulating EGFR signalling.

Analysis of the cancer genome atlas (TCGA) database, a landmark cancer genomics programme that molecularly characterized over 20 000 primary cancer and matched normal samples spanning 33 cancer types, indicates that DHHC20 is highly expressed in breast and lung cancer cell lines where EGFR signalling is biologically relevant [21]. Initial hints that palmitoylation may regulate EGFR signalling came from experiments that showed that silencing the palmitoyltransferase DHHC20 in the triple-negative breast adenocarcinoma line, MDA-MB-231, enhanced the duration and amplitude of EGFR activation and signalling [21]. While EGFR is already membrane-localized we reasoned that DHHC20 might regulate EGFR by palmitoylating EGFR itself. To test this possibility, we performed acyl-biotin exchange (ABE) assays to detect palmitoylated endogenous proteins [27]. The ABE assay substitutes biotin for palmitate on cysteine residues, allowing enrichment by isolation on streptavidin beads. EGFR was readily detected as a palmitoylated protein in these assays [21]. Stimulating EGFR with the canonical ligand EGF further enhanced EGFR palmitoylation, suggesting that palmitoylation follows EGFR activation [21]. EGFR binds to six other peptide growth factor ligands that result in different amplitudes and durations of receptor activation, and it will be interesting to know if they have different effects on EGFR palmitoylation [28].

To further validate that EGFR is palmitoylated, we employed a metabolic labelling approach. We metabolically labelled cells with a functionalized palmitate analogue, palmitic acid azide. When we enriched proteins that incorporated palmitic acid azide by click chemistry, we detected endogenous EGFR, again confirming that EGFR is palmitoylated [21]. Moreover, loss of endogenous DHHC20 in these cells reduced EGFR palmitoylation, suggesting that DHHC20 palmitoylates EGFR [21]. While this does not rule out that DHHC20 may palmitoylate a direct or indirect regulator of EGFR signalling, these experiments are strong evidence that EGFR is a substrate of DHHC20 in mammalian cells.

Of note, the fraction of palmitoylated EGFR relative to total EGFR detected in these assays is relatively low (typically less than 10%), and the increase in EGFR palmitoylation in response to EGF stimulation is relatively modest. As such, it is intriguing that a relatively small change in EGFR palmitoylation upon loss of DHHC20 is associated with such a drastic change in the amplitude and duration of EGFR signalling. By contrast to what might be expected in the case of enhanced EGFR signalling, the loss of DHHC20 markedly increased EGFR ubiquitylation, a marker of EGFR signal termination which facilitates endosomal trafficking to the lysosome for degradation [21]. However, lysosomal targeting of EGFR in these cells was perturbed, and internalized EGFR remained ‘trapped’ in endosomes with Grb2 where it is thought that EGFR can continue to signal [21]. Overall, EGFR palmitoylation appears to be important for receptor turnover which may explain the large impact on signal activation when palmitoylation of a small pool of receptor is blocked.

How might a reduction in EGFR palmitoylation enhance EGFR signalling? To answer this question, we identified the location of the palmitoylation site(s) in EGFR. Rigorous identification of palmitoylated residues is technically challenging and, therefore, is often not performed for most studies of palmitoylated proteins. We identified the specific palmitoylated sites on EGFR to provide insight into how palmitoylation of the tail inhibits signalling. We used the ABE assay to purify and differentially label palmitoylated and free cysteine residues in EGFR using tags with unique molecular weights that could be distinguished by mass spectrometry [21]. These experiments indicated that multiple cysteines in the CTD of EGFR, including C1025 and C1034, are palmitoylated. Mutation analysis suggests C1122 may also be palmitoylated, but C1122 is located in a peptide that is too large to be analysed by standard mass spectrometry methods [21]. The CTD has been suggested to play an autoinhibitory role in EGFR activation by a yet-unclear mechanism, raising the interesting possibility that palmitoylation of C1025 and C1034 may contribute to this mechanism [12,29]. To examine this further, we generated palmitoylation-deficient mutants by mutating the palmitoylated cysteine residues to alanine (C1025A and C1034A). Although we were unable to express the C1034A EGFR-mutant in mammalian cells, the C1025A EGFR-mutant expressed well and localized to the cell membrane similarly to wild-type EGFR [21]. Expression of the C1025A EGFR-mutant enhanced EGFR activation as measured by EGFR and ERK phosphorylation, both in response to EGF and also in a ligand-independent manner [21]. We also found that the C1025A EGFR-mutant had increased association with Grb2, a scaffold protein that binds to the phosphorylated CTD of activated EGFR, compared to wild-type EGFR [21]. This suggests that palmitoylation of C1025 might preclude Grb2 binding possibly through either a direct steric effect or palmitoylation-induced conformational changes in the CTD. These findings support the idea that palmitoylation of C1025 is an important component of the regulatory role of the EGFR CTD. Future studies on the temporal regulation of CTD palmitoylation will resolve the relative contributions of the other putative palmitoylation site(s) in the CTD and whether multiple sites are palmitoylated simultaneously and how such a process is regulated.

### 2.4. Recognition of EGFR by DHHC20

The mechanism by which DHHC family palmitoyltransferases recognize substrates remains unclear, and consensus palmitoylation sequence motifs have yet to be identified. Although EGFR is palmitoylated by DHHC20, remarkable conservation in the sequence of the catalytic domain and observed substrate promiscuity among the DHHC family members suggests that other DHHC enzymes can palmitoylate EGFR. However, expression patterns of the DHHC enzymes and changes in their expression levels in different cancers are tissue-specific. In the case of EGFR regulation in lung and breast cancers, DHHC20 is likely to be the physiologically relevant DHHC enzyme, given that DHHC20 is expressed in multiple lung and breast cancer cell lines [21]. Therefore, for the remainder of this section, we will focus on molecular recognition of EGFR by DHHC20, the DHHC family member most biologically relevant to EGFR regulation in lung and breast cancer.

How might DHHC20 recognize and palmitoylate EGFR? Given that both DHHC20 and EGFR localize at the plasma membrane, it is plausible that spatial restriction contributes to substrate specificity. In addition to spatial constraints, steric constraints are likely to play a role in controlling EGFR palmitoylation by DHHC20. Based on the crystal structures of DHHC20, the only DHHC family member structurally characterized to date, substrate approach to the active site of DHHC20 is limited to one surface due to structural constraints on the catalytic domain [30]. This surface has a positively charged surface potential, suggesting that electrostatic interactions between the substrate and enzyme may contribute to substrate recognition. It is possible that palmitoylation follows EGFR activation and tyrosine phosphorylation of the CTD, although it is currently unclear if DHHC20 exhibits a preference for phosphorylated or unphosphorylated CTD. Therefore, such an interaction may be mediated by negatively charged residues in the tail or phosphotyrosine-rich regions in the CTD of the activated receptor. The contribution of the region directly proximal to the active site to substrate recognition is not well established, however, and the dynamics of DHHC20 that may contribute to substrate binding and release are not revealed by the static structure solved by crystallographic studies. Moreover, while the CTD of EGFR is the site of palmitoylation, other domains of EGFR may contribute to substrate recognition by DHHC20. Additionally, the interaction between EGFR and DHHC20 may be promoted by the binding of a secondary ligand or protein complex. Ultimately, the establishment of physiologically relevant DHHC enzyme–substrate pairs may be resolved by *in vivo* loss of function studies using genetic mouse models.

### 2.5. EGFR palmitoylation: shedding light on enigmatic aspects of EGFR signalling

Evidence implicating the CTD of EGFR in receptor inhibition appears in numerous biochemical and genetic studies, including the identification of activating deletions in the CTD in glioblastoma patients [12,29,31]. Does palmitoylation of the CTD provide a mechanism for the observed autoinhibitory function of the CTD? The complete CTD has not been observed in any EGFR structures to date, although a fragment of the CTD has been observed bound to the kinase domain in X-ray crystal structures of the inactive kinase domain [12]. These structures suggested that interactions between the kinase domain and CTD may regulate EGFR activation, but have not been functionally validated. Mutagenesis of the CTD has also suggested that the portion of the CTD which contains the C1025A palmitoylation site is autoinhibitory. Deletion of the proximal CTD (residues 958–1030, termed the ΔvIVb variant identified in glioblastoma multiforme patients), which removes the C1025 palmitoylation site, enhances EGFR signalling as measured by phosphorylation of the downstream MAPK effector Erk [29,32]. This is consistent with our observation that expression of the C1025A mutant of EGFR enhances EGFR signalling and Erk phosphorylation. Along these lines, deletion of the region proximal to C1025 (residues 958–1005) and including C1025 (983–1028) have the strongest activating effects on EGFR signalling [29]. While deletion of residues 958–1005 does not remove C1025, it is possible that C1025 is no longer accessible for palmitoylation when the proximal region is deleted. Overall, multiple lines of evidence support the idea that C1025 palmitoylation may contribute to the autoinhibitory function of the CTD.

**Figure 1.**
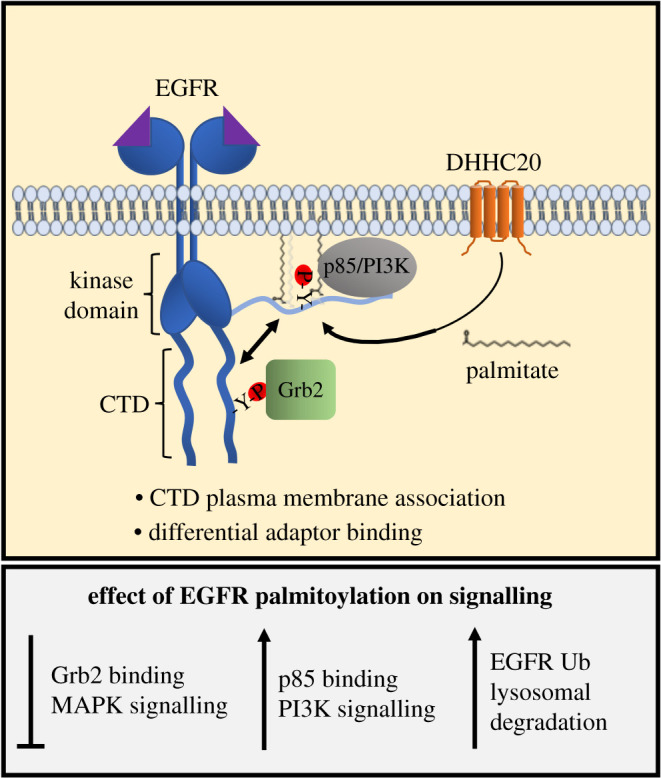
Model of signal regulation by EGFR palmitoylation. Ligand binding to EGFR causes receptor dimerization and autophosphorylation of tyrosine residues on the C-terminal domain (CTD). DHHC20 palmitoylates EGFR on the CTD adjacent to phospho-tyrosine residues. Palmitoylation decreases association of the adaptor protein Grb2 that mediates MAPK signalling and increase association with p85 the regulatory subunit of the PI3K signalling complex. Evidence suggests palmitoylation may increase the association of the CTD with the plasma membrane. Reducing EGFR palmitoylation increases total levels of receptor and decreased trafficking to the lysosome.

Given that palmitoylation typically promotes membrane association of cytosolic proteins, one possibility is that palmitoylation increases the association of the CTD with the plasma membrane, sequestering the CTD away from the kinase domain, preventing activating autophosphorylation (figure 1). In support of this model, we found that the CTD associated with the isolated membrane fraction after the release of the CTD by proteolytic cleavage [21]. One might envision that decreased accessibility of the CTD by association with the plasma membrane may negatively regulate EGFR signalling by precluding Grb2 binding, favouring the binding of other adaptors to promote signalling pathways parallel to MAPK [21] (figure 1). This mechanism may be dictated by the intrinsically disordered nature of the C-terminal tail, for example. The hydrodynamic radius, or capture radius, of the tail may decrease after being pinned to the membrane by palmitate, favouring an interaction with membrane-associated proteins such as PIK3R1, the regulatory subunit of phosphoinositide 3-kinase (PI3K). This ‘fly casting’ mechanism has been described for the regulation of other proteins with intrinsically disordered domains by post-translational modifications [33].

Palmitoylation may specify different signalling kinetics in response to unique EGFR ligands. EGFR is known to bind at least seven different extracellular ligands, which produce either sustained or transient EGFR activation and signalling [34]. Specification of the response to each ligand has been suggested to be influenced by the kinetics of ligand binding and post-endocytic trafficking, whereby ligands that promote more short-lived EGFR dimers elicit sustained signalling responses [34,35]. This can be explained by the concept of ‘kinetic proofreading’, where only the longest-lived EGFR dimers can trigger negative feedback to activate negative regulatory mechanisms [34,35]. The finding that the loss of DHHC20 and EGFR palmitoylation in MDA-MB-231 breast cancer cells and SW1573 lung cancer cells extended the duration of EGFR signalling in response to EGF highlights a possible connection between EGFR palmitoylation and EGFR signalling kinetics [21]. It is plausible that the levels or kinetics of EGFR palmitoylation differ with different EGFR ligands, influencing signalling kinetics. Dissecting how EGFR palmitoylation changes in response to different EGFR ligands, and how palmitoylation influences post-endosomal EGFR trafficking, will provide insight into the impact of palmitoylation on receptor kinetics and formation of receptor homo- or heterodimers, or if palmitoylation regulates post-endosomal EGFR trafficking in response to different ligands.

Overall, EGFR palmitoylation appears to be a previously unknown contributor to EGFR signalling regulation. Several components that have been missing from our understanding of EGFR signalling, such as information about the role of the CTD in signalling and differential responses to extracellular ligands as highlighted above, may involve EGFR palmitoylation.

## 3. EGFR palmitoylation as a potential therapeutic vulnerability in cancer

### 3.1. EGFR palmitoylation is required for tumorigenesis in a genetically engineered mouse model of lung cancer

**Figure 2.**
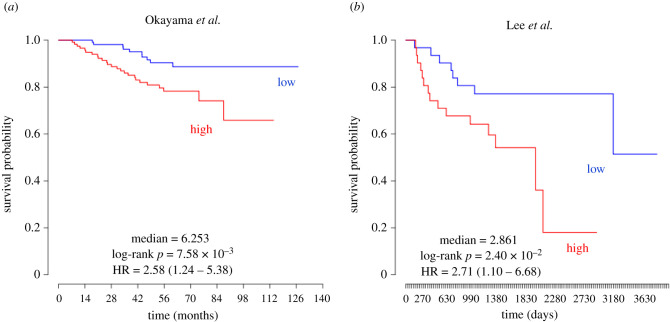
Kaplan–Meier plots generated by the PRECOG portal showing survival probability over time from lung cancer patients with high (red) or low (blue) ZDHHC20 expression [36]. (*a*) The study by Okayama *et al*. shows a hazard ratio = 2.58 (1.24–5.38) and a log-rank *p* = 7.58 × 10^−3^ [37]. (*b*) The study by Lee *et al*. shows a hazard ratio = 2.7 (1.10–6.68) and a log-rank *p* = 2.40 × 10^−2^ [38].

Although blocking EGFR palmitoylation hyperactivates EGFR, there are relatively few examples in the literature implicating DHHC enzymes in the initiation or progression of cancer. The PRECOG (prediction of clinical outcomes from genomic profiles) portal allows the query of associations between genomic profiles and cancer outcomes [36]. Expression analysis of ZDHHC20 mRNA from lung adenocarcinoma patients using the PRECOG portal correlates high ZDHHC20 mRNA expression with lower survival probability (*Z*-score 3.19). Two individual studies included in the PRECOG analysis (by Okayama *et al*. [37] and Lee *et al*. [38]) showed clear improved survival probability in patients with low ZDHHC20 expression (figure 2*a*,*b*). This would suggest that reducing ZDHHC20 *in vivo* should have a negative effect on either tumour growth or progression, although it is not apparent from these studies if these effects stem from a loss of EGFR palmitoylation by DHHC20. Another study performed massive parallel sequencing on 183 lung adenocarcinoma patients and identified complex genomic rearrangements that generated a novel deletion of exons 25 and 26 in the CTD of EGFR [39]. The deletion caused activation of the EGFR/AKT signalling axis, and cellular transformation as measured by anchorage-independent growth of cultured cells [39]. While the mechanism of EGFR activation by this deletion mutant was unknown, we reasoned that EGFR activation may be caused by the deletion of palmitoylated cysteine 1025 in exon 26. In total, these studies make lung cancer an optimal model to test the impact of palmitoylation inhibition on tumorigenesis *in vivo*.

To address the function of EGFR palmitoylation in lung cancer, we used an autochthonous genetically engineered mouse model of lung adenocarcinoma driven by oncogenic Kras^G12D^; in this mouse model, the progression to adenocarcinoma is accelerated by deletion of the tumour suppressor p53 [40,41]. To simultaneously inactivate *Zdhhc20* gene expression while activating *Kras^G12D^*, the mice were transduced with a lentiviral construct expressing Cre recombinase, Cas9 and a single-guide RNA (sgRNA) targeting *Zdhhc20* (shDHHC20) or a non-targeting control (sgControl) [40,41]. Viral infection results in tissue-specific ablation of *Zdhhc20* and Cre-induced expression of YFP and *Kras^G12D^*, and deletion of p53 [40,41].

Ablation of the *Zdhhc20* gene caused a potent 10-fold reduction of tumour formation compared to sgControl mice [40]. This effect was durable, as after 24 weeks tumours still had not formed in the sgDHHC20-expressing mice while the sgControl mice had to be sacrificed at 12 weeks because of their severe tumour burden. We identified the virus-infected, YFP-positive lung cells by immunohistochemistry and found that a lower percentage of sgDHHC20-infected cells expressed the cell proliferation marker Ki67 than tumours infected with control vectors, indicating that DHHC20 loss did not induce cell death but instead blocked cell proliferation [40]. Moreover, consistent with what we observed in the human lung cancer cell lines, the level of phosphorylated ERK was increased in sgDHHC20-infected tumours compared to those infected with sgControl. This study conclusively showed a requirement for DHHC20 in Kras-mutant lung adenocarcinoma for tumour initiation and cell proliferation.

To confirm that the block in tumour formation was caused by loss of EGFR palmitoylation we expressed the palmitoylation-defective EGFR^C1025A^ mutant in the same mouse model. Mice expressing EGFR^C1025A^ failed to form tumours similar to what we observed in the *Zdhhc20*-ablated mice. Reducing tumour formation while increasing EGFR activation seems counterintuitive. However, our results are similar to observations by other groups showing that activating mutations in EGFR and in Kras are synthetically lethal [42,43]. Consistent with the synthetic lethal mechanism, the deletion in exons 25 and 26 that removes palmitoylated cysteine 1025 in lung cancer patients was also found to be mutually exclusive from activating Kras mutations [30].

### 3.2. EGFR palmitoylation-dependent signalling required for lung cancer proliferation

To identify the changes in mitogenic signalling pathways that inhibit tumour growth in the mouse model, we transiently knocked down DHHC20 in an oncogenic Kras-harbouring lung adenocarcinoma cell line NCI-H23 and examined the differences in signalling downstream of EGFR [40]. DHHC20-deficient NCI-H23 cells displayed reduced cell proliferation decreased AKT (T308) and GSK3β (S9) inhibitory phosphorylation leading to Myc phosphorylation and proteosomal degradation of Myc protein, suggesting that PI3K–AKT signalling, an EGFR downstream pathway parallel to the MAPK pathway, might play a role in the regulation of palmitoylation-dependent tumour growth. Treating DHHC20-deficient NCI-H23 cells with the proteasome inhibitor MG132 or GSK3β inhibitor CHIR-9002 partially restored Myc protein levels, indicating the direct involvement of GSK3β-directed Myc proteasomal degradation in palmitoylation-dependent tumour growth [40]. Expression of stabilized Myc^T58A^, a mutant unable to be targeted for proteosomal degradation by GSK3β-mediated phosphorylation, in DHHC20-deficient NCI-H23 cells fully restored the growth rate to that of the control cells, suggesting that the growth inhibiotin of palmitoylation-defective NCI-H23 cells was caused by reduced Myc expression [40].

**Figure 3.**
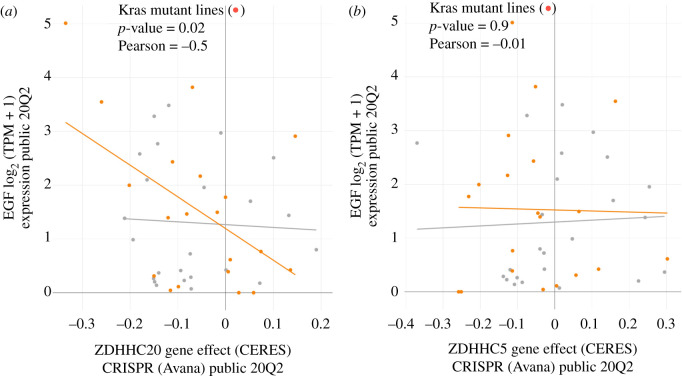
Graphs generated by the DepMap portal showing the cell growth effect of CRISPR mediated ablation of ZDHHC20 or ZDHHC5 in Kras-mutant lung cancer cell lines plotted against the mRNA expression of an EGFR ligand EGF. (*a*) The negative cell growth with ZDHHC20 CRISPR correlates with EGF expression only in Kras-mutant cell lines suggesting a correlation with EGFR activation (*p*-value = 0.02, Pearson = −0.5). (*b*) This correlation is not observed with ZDHHC5 CRISPR (*p*-value = 0.9, Pearson = −0.01).

Similar inhibition of xenograft tumour growth was observed by another group upon reduction of the palmitoyl transferase DHHC5 [44]. The reduction of tumour growth upon genetic inhibition of either DHHC5 or DHHC20 suggests these transferases are not redundant and the pathways downstream of each enzyme are likely to be unique. While the mechanism of DHHC5 function in NSCLC is still unknown it is possible to compare the effect of ablating DHHC20 with DHHC5 in a large dataset from human NSCLC cell lines in the Cancer Dependency Map (DepMap) database to correlate the dependence of the cell growth effect with expression or mutation of EGFR signalling components [45]. Analysis of the Avana CRISPR screen of NSCLC adenocarcinoma cell lines where DHHC20 was ablated indicates the negative effect on growth is correlated with expression of the EGFR ligand EGF in cell lines expressing oncogenic Kras (figure 3*a*). By contrast, we were unable to find a correlation with expression of EGF expression in Kras mutant or wild-type cell lines when DHHC5 is ablated (figure 3*b*). Taken together these data suggest the context in which DHHC5 is required for cancer cell growth is distinct from that of DHHC20.

In sum, EGFR palmitoylation appears to be required for PI3K–AKT–Myc signalling in Kras-mutant lung adenocarcinoma, revealing DHHC20 inhibition as a therapeutic vulnerability in the PI3K–AKT pathway.

### 3.3. The clinical relevance of EGFR palmitoylation in cancer therapeutics

Currently, there are no small-molecule inhibitors specific to DHHC enzymes. The inhibitor 2-bromopalmitate (2-BP) is a non-metabolizable palmitate analogue widely used as a research tool for studying palmitoylation. However, 2-BP has limited potential as a therapeutic lead compound because it exhibits low specificity to any one DHHC enzyme [46]. More, recently, developed inhibitors, such as cyano-myracrylamide (CMA), exhibit improved properties over 2-BP, but are not specific to a single DHHC enzyme [47]. There has been little incentive to develop selective inhibitors of DHHC enzymes because so few diseases have been linked to DHHC enzyme function. Our discovery that genetically ablating DHHC20 almost completely blocks Kras-driven lung adenocarcinoma formation suggests DHHC could be a possible therapeutic target since there are still very few effective treatment strategies for Kras-driven cancers.

Ablating the *Zdhhc20* gene effectively blocked tumour formation in the genetically engineered mouse model but demonstrating that blocking DHHC20 potently inhibits the growth of existing tumours is more relevant to its potential as a therapeutic strategy. We therefore generated a Kras-mutant human lung cancer cell line that expresses DHHC20 shRNA in response to treatment with doxycycline and implanted the cells in the flanks of immunocompromised mice (SCID) [40]. Induction of the DHHC20 shRNA with doxycycline significantly inhibited the growth of tumours by day 2 of treatment compared to tumours expressing control shRNA [48]. The ability of acute DHHC20 inhibition to block the growth of tumours established from human lung cancer cells strengthens the rationale for targeting DHHC enzymes as potential therapeutic targets.

While TKIs have revolutionized the treatment of oncogenic tyrosine kinase-driven malignancies, the use of TKIs in the clinic has been challenging. Although lung cancer patients harbouring activating mutations in EGFR display initial response to TKIs targeting the activating mutation, most patients acquire resistance after a progression-free period of about 10 months due to secondary mutations in the kinase domain of EGFR [49]. Therefore, other mechanisms of regulating EGFR signalling are potential vulnerabilities in multiple cancer types, either targeted alone or in combination with existing therapies. For example, the lung adenocarcinoma cell line NCI-H1975 harbours both the primary activating L858R mutation that increases the affinity for the EGFR inhibitor gefitinib and the secondary T790M mutation that imparts resistance to gefitinib [50]. As such, the NCI-H1975 lung adenocarcinoma cell line is relatively resistant to gefitinib. We found that silencing DHHC20 in NCI-H1975 cells increased EGFR activation and signalling to Erk, but also enhanced sensitivity to gefitinib-induced growth inhibition compared to control shRNA expressing cells. The combined treatment of NCI-H1975 with 2-BP and gefitinib resulted in a synergistic block in cell growth compared to either inhibitor alone [48]. These results not only demonstrate that DHHC20 is a druggable target for cancer therapy but also provide a novel way to sensitize gefitinib-resistant lung cancer to a small-molecule TKI.

A similar vulnerability in the PI3K–AKT signalling pathway in Kras-mutant cancer growth was also discovered. Reduction of DHHC20 expression by shRNA in the Kras-mutant lung cancer cell line SW1573 led to an increase in pan-PI3K inhibitor BKM120 (buparlisib)-induced cell death [40]. Moreover, DHHC20 knockdown in both SW1573 and NCI-H23 cells decreased the IC_50_ concentrations of BKM120, suggesting that loss of DHHC20 sensitizes Kras-mutant lung cancers to PI3K inhibitors [40]. Whether this is caused by decreased palmitoylation of EGFR or an alternate DHHC20 target remains to be determined.

In summary, these findings provide a strong rationale for the development of small-molecule DHHC20 inhibitors, and the development of a combined regimen to treat Kras-mutant cancers with a DHHC20 inhibitor and a PI3K inhibitor.

## 4. Final remarks

The question remains if palmitoylation is a broadly conserved mechanism for receptor tyrosine kinase regulation. In addition to EGFR, we have found that the other ErbB family members are palmitoylated, yet we have not determined whether the location and function are also conserved with EGFR. Palmitoylation has been found to regulate signalling processes of other RTK family members. A recent study determined that palmitoylation regulates the intracellular trafficking and stability of the Met receptor tyrosine kinase which plays a critical role in various biological phenomena, including embryonic development, organ regeneration, tumorigenesis and metastasis [51,52]. The Met protein has a unique structure as it is synthesized as a 170 kD single-chain precursor that is subsequently glycosylated and cleaved in the Golgi apparatus into a 50 kD α-chain and a 140 kD β-chain that are linked by disulfide bond [53]. Upon the binding of hepatocyte growth factor, Met receptor is activated by dimerization and *trans*-phosphorylation of two catalytic tyrosine residues (Y1234 and Y1235) within the kinase activation loop, followed by phosphorylation of two docking tyrosine residues (Y1349 and Y1356) located in the C-terminal tail, ultimately recruiting adaptor proteins and activating downstream MAPK, JNKs, PI3K–AKT and STATs signalling pathways [52]. S-palmitoylated Met has been detected in multiple cancer cell lines, including prostate, lung and breast cancer. Coleman *et al.* [51] reported that inhibiting palmitoylation with 2-BP reduced Met expression and disrupted the trafficking of Met in prostate cancer cell line DU145. Through metabolic labelling and mutation of all β-chain cysteine residues not involved in disulfide bonding, they identified two potential palmitoylation sites (C894 and C624) located on the extracellular domain of Met β-chain [51]. Their findings indicated that palmitoylation is essential for the transportation of mature Met to the plasma membrane which could be a potential vulnerability in Met-driven cancers.

In addition to lung cancer, our laboratory also demonstrated the function of DHHC20 in breast cancer cell line MDA-MB-231 which has elevated levels of wild-type EGFR [21]. When DHHC20 expression is reduced with shRNA, cells display an increased migratory ability which is consistent with the well-described function of EGFR in promoting cell migration. Knowing that DHHC20 ablation would sensitize cancer cells to small-molecule TKI and that MDA-MB-231 cells express unusually high levels of wild-type EGFR, we treated these cells with gefitinib after DHHC20 disruption and found an elevated gefitinib-induced cell death in DHHC20 shRNA expressing cells compared to control shRNA expressing cells [21]. To test whether unpalmitoylated EGFR is the main mechanism that increases TKI sensitization in breast cancer, we expressed a tetracycline-inducible palmitoylation-defective EGFR (EGFR^C1025A^) in MDA-MB-231 cells that led to increased cell death after gefitinib treatment compared to those expressing inducible wild-type EGFR, suggesting that the sensitization of triple-negative breast cancer cells to gefitinib is mediated by unpalmitoylated EGFR [48,54].

While DHHC20 is important for EGFR palmitoylation and Kras-driven lung cancer formation, we have not ruled out the possibility that other DHHC enzymes may also palmitoylate EGFR or other RTKs. It is, therefore, important to note recently identified roles for DHHC enzymes in suppressing or promoting cancer through mechanisms that have yet to be elucidated and could potentially involve RTKs. DHHC2 has been proposed as a putative tumour suppressor gene because the low levels of DHHC2 expression in patient samples are associated with lymph node metastasis and poor prognosis in gastric adenocarcinoma [54]. Similarly, overexpression of DHHC14 inhibits tumorigenesis in a mouse xenograft model, functionally implicating it as a tumour suppressor [55]. Finally, elevated expression of DHHC3 promotes breast tumour growth *in vivo*, while ablating DHHC3, reduces *in vivo* tumour growth and induces oxidative stress and senescence [56]. It is worth noting that there are also increasing examples of DHHC enzymes and their substrates regulating cancer initiation or progression. For example, DHHC5-mediated palmitoylation of the histone methyltransferase EZH2 is associated with malignant development and progression of glioma harbouring mutant p53 and DHHC13-dependent palmitoylation of the melanocartin-1 receptor (MC1R), an important G-protein-coupled receptor in human and mouse pigmentation, triggers senescence and protection against melanomagenesis [57,58].

These emerging studies implicate DHHC enzymes in tumorigenesis or tumour progression under different cellular contexts, consolidating the idea of targeting DHHC acyltransferases as a mono- or combined cancer therapy, or cancer prevention strategy. DHHC acyltransferases have been found to palmitoylate hundreds of mammalian proteins, suggesting that the DHHC enzymes may regulate multiple cellular signalling mechanisms that extend beyond EGFR palmitoylation. There are multiple strategies for implementing DHHC inhibitors, but another major step is identifying molecular classifications of tumours that are most likely to respond to future inhibitors and what factors will predict positive patient outcome.

## Data accessibility

This article has no additional data.
